# The roles of connexins and gap junctions in the progression of cancer

**DOI:** 10.1186/s12964-022-01009-9

**Published:** 2023-01-13

**Authors:** Mingming Zhou, Minying Zheng, Xinyue Zhou, Shifeng Tian, Xiaohui Yang, Yidi Ning, Yuwei Li, Shiwu Zhang

**Affiliations:** 1 Graduate School, Tianjin Medical University, Tianjin, 300070 People’s Republic of China; 2 Department of Pathology, Tianjin Union Medical Center, Nankai University, Tianjin, 300121 People’s Republic of China; 3 Nankai University School of Medicine, Nankai University, Tianjin, 300071 People’s Republic of China; 4 Department of Colorectal Surgery, Tianjin Union Medical Center, Tianjin, 300121 People’s Republic of China

**Keywords:** Gap junctions, Connexins, Post-translational modification, Cancer, Epithelial–mesenchymal transition

## Abstract

Gap junctions (GJs), which are composed of connexins (Cxs), provide channels for direct information exchange between cells. Cx expression has a strong spatial specificity; however, its influence on cell behavior and information exchange between cells cannot be ignored. A variety of factors in organisms can modulate Cxs and subsequently trigger a series of responses that have important effects on cellular behavior. The expression and function of Cxs and the number and function of GJs are in dynamic change. Cxs have been characterized as tumor suppressors in the past, but recent studies have highlighted the critical roles of Cxs and GJs in cancer pathogenesis. The complex mechanism underlying Cx and GJ involvement in cancer development is a major obstacle to the evolution of therapy targeting Cxs. In this paper, we review the post-translational modifications of Cxs, the interactions of Cxs with several chaperone proteins, and the effects of Cxs and GJs on cancer.

**Video Abstract**

**Supplementary Information:**

The online version contains supplementary material available at 10.1186/s12964-022-01009-9.

## Introduction

Gap junctions (GJs) are specialized protein channels that allow two neighboring cells to communicate directly with each other. This process is called gap junction intercellular communication (GJIC). The function of GJIC is achieved by gap junctions (GJs). The main components of GJs are connexins (Cxs).

GJIC is widely distributed between normal–normal, cancer–cancer, and cancer–normal cells. Substance exchange between normal microvascular endothelial cells and cancer-associated fibroblasts (CAFs) can regulate cellular proliferation [1], the cell cycle itself [2, 3], the expression of epithelial–mesenchymal transition (EMT)-related proteins [4–6], self-renewal of cancer cells [7] and angiogenesis [8]. In addition, the exchange of information between cancer cells via GJIC is associated with the anti-tumor effects of chemical reagents [9]; cytotoxic substances, such as reactive oxygen species (ROS) produced by radiotherapy, are transmitted through GJIC [10, 11]. The balance between the synthesis and degradation of Cxs and GJs is critical for maintaining homeostasis in normal cells. GJIC and Cxs not only play an important role in normal physiological processes, such as embryonic development [8, 12, 13], bone formation [14, 15], and ovum expulsion [16], but are also associated with some pathological processes, including wound healing [15] and inflammatory responses [17].

The relationship between GJIC and cancer has been receiving increasing attention. Cxs have been characterized as tumor suppressors in the past, with the main evidence thereof being as follows: (1) Tumor cells lack functional GJIC, and cancer cells do not express Cxs in HeLa28 [18] and MCF-7 cancer cells [19]; (2) Tumor-promoting chemicals and conditions reversibly inhibit GJIC; (3) Some oncogenes, including *src*, *ras*, *raf*, and *mos*, can decrease the expression of GJIC; (4) Cx gene transfection inhibits the growth and decreases the tumorigenicity of tumor cells [19, 20]; (5) Mice with connexin-32 knockout can develop spontaneous and chemically induced liver cancer [20]. However, recent studies have revealed that Cxs and GJs in fact play crucial roles in cancer pathogenesis. GJs can increase tumor cell invasion and migration in EMT-dependent and EMT-independent pathways. Abnormalities in the function of GJIC or the expression levels of Cxs are generally also accompanied by the onset of cancer. In addition, GJIC and Cxs are associated with the grade and stage of cancer [21–24], with abnormal expression and subcellular localization of Cxs being associated with cancer initiation and progression. Post-translational modifications (PTMs) of proteins, which occur via phosphorylation, acetylation, ubiquitination, and SUMOylation, can regulate their active state and subcellular localization. In this review, we discuss the PTMs of Cxs, the interaction of Cxs with several chaperone proteins, and the effects of Cxs and GJs on cancer.

### Structure and function of Cxs

**Fig. 1:**
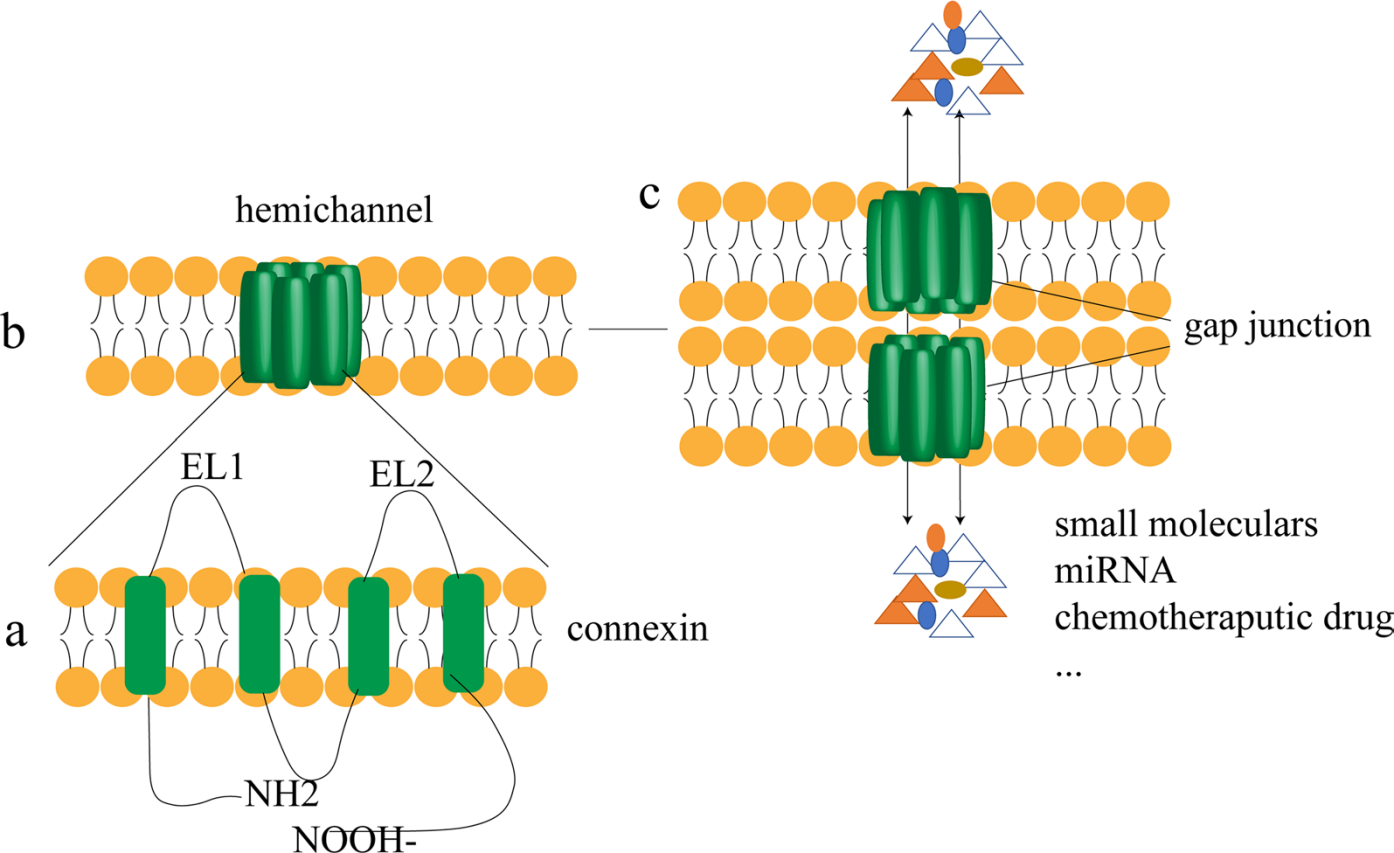
**a** Structure of connexins (Cxs). Cxs contain a cytoplasmic N-terminus, four transmembrane domains, two extracellular loops (EL1 and EL2), a cytoplasmic loop, and a cytoplasmic carboxyl terminus tail. **b** Six Cxs assemble into a hemichannel, which is a hollow structure. **c** Hemichannels of two adjacent cells connect in a head-to-head manner to form gap junctions (GJs). The exchange of various substances mediated by GJs is called gap junction intercellular communication (GJIC)

To date, 21 Cxs have been identified. The isoforms of Cxs are named according to their molecular weights and order of discovery, and each Cx has its own coding gene. The distribution of each isoform is also tissue- or cell-specific (Table 1). All Cxs are tetraspanins with a cytoplasmic N-terminus, two outer membrane loops, an inner membrane loop, and a carboxyl-terminus located in the cytosol. The two outer membrane loops of Cxs, denoted by EL1 and EL2, are conserved regions (Fig. 1). Six Cx monomers can be assembled into a cyclic hexameric hemichannel. Two hemichannels located on the membranes of different cells form an intact GJ in a head-to-head manner. Several or even thousands of GJs can form GJ plaques. GJs undergo dynamic changes that can be updated continuously [25]. The turnover of GJ plaques generally occurs when hemichannels accumulate in the periphery of GJ plaques and form new GJs, whereas GJs present inside GJ plaques are first internalized and then degraded [26]. GJs are selective for substance delivery; only small molecules with molecular weights less than 1 × 10^3^, such as inorganic salts, glucose, prostaglandins, and secondary messengers, can pass through them. Mitochondria [27], microRNAs (miRNAs) [8], and certain chemotherapeutic drugs such as gemcitabine [9] have also been reported to pass through GJs. The exchange of substances mediated by GJIC is bidirectional and, in a few cases, unidirectional [6, 28].

**Table 1:** Coding genes and tissue distribution of connexins (Cxs)

Gene	Cx	Expression in normal human tissue
*GJA1*	Cx43	Cardiac [29], lung [30], skin [31]
*GJA3*	Cx46	Cartilage [32], lens [33]
*GJA4*	Cx37	Spinal cord [34], ovary [35]
*GJA5*	Cx40	Spinal cord [34]
*GJA8*	Cx50	Lens [33]
*GJA9*	Cx59	Retina [36]
*GJA10*	Cx62	Platelets [37]
*GJB1*	Cx32	Liver [38, 39], cartilage [32], oligodendrocytes [40]
*GJB2*	Cx26	Liver [38, 39], astrocytes [40], cochlea [41]
*GJB3*	Cx31	Cochlea [41]
*GJB4*	Cx30.3	Thymus [42]
*GJB5*	Cx31.1	Trophoblast cells [43], epidermis [44, 45]
*GJB6*	Cx30	Astrocytes [46]
*GJB7*	Cx25	Hematopoietic stem cells [47]
*GJC1*	Cx45	Cardiac cells [48]
*GJC2*	Cx47	Astrocytes [49], oligodendrocytes [49]
*GJC3*	Cx30.2/31.3	Oligodendrocytes [50]
*GJD2*	Cx36	Retina [51]
*GJD3*	Cx31.9	Cardiac cells [52]
*GJD4*	Cx40.1	Skeletal muscle [53]
*GJE1*	Cx23	Lens [54]

The first two columns were compiled from NCBI (National Center for Biotechnology Information (nih.gov)) data. Currently, 21 types of Cxs are known. The expression of each type of Cx in the human body is spatially different. The third column provides examples of the distribution of Cxs in normal human tissue

### Regulatory mechanisms related to the expression and function of Cxs and GJs

#### Positive regulation of Cxs and GJs

The expression of Cxs, opening and closing of GJ gating, and size of GJ plaques play decisive roles in intercellular material exchange [55]. Various intracellular proteins and cytokines are involved in regulating Cxs and GJs. Cx monomers are first synthesized in the endoplasmic reticulum and then transported to the Golgi apparatus to form hexameric structures [56–58]. These structures are transported by vesicles to the cell membrane surface for assembly into GJs [59]. For example, the TGF-β2/Smad3 pathway promotes Cx43 synthesis in the endoplasmic reticulum [60]. Connexin 43-interacting protein of 75 kDa (CIP75), belonging to the ubiquitin-like and ubiquitin-associated domain family of proteins, promotes the proteasome-mediated degradation of Cx43 monomers located on the endoplasmic reticulum [61]. In addition, the interaction between CIP75 and Cx43 promotes the transfer of Cx43 from the endoplasmic reticulum to the Golgi apparatus, which releases Cx43 from its “binding” to the endoplasmic reticulum [61]. Cx43 is transported to the cell membrane by vesicles after its assembly into hemichannels. This transport process occurs along microtubules. Studies have shown that the combination of Cx43 and α/β-tubulin ensures the movement of Cx43 to the cell membrane and also helps to maintain the stability of microtubules, which is important for the maintenance of cell polarity and homeostasis [62–64]. Phosphorylation of Cx43 at Tyr247 hinders the binding of Cx43 to tubulin [65]. Interestingly, Yun Fu et al. [66] showed that Cx43 overexpression inhibits α/β-tubulin expression in MDA-MB-231 cells. In addition to tubulin, F-actin facilitates the transport of hemichannels to the cell membrane. However, F-actin and Cx43 cannot bind directly, and drebrin is required as a mediator [67]. Zonula occludens-1 (ZO-1) protein mediates hemichannel assembly in cell membranes and its overexpression increases the number of GJ plaques on the cell membrane while enhancing the transport capacity of substances. It also increases the size of the plaques compared to that of control cells [25, 68]. Disruption of the junction between Cx and ZO-1 does not significantly change the amount of Cx43 on the cell membrane but drastically decreases the ability of GJs to exchange substances [68]. This implies that ZO-1 not only affects the formation of GJs from Cxs but also has important implications for GJIC function. The half-life of Cx43, which does not detach from ZO-1 after binding to it, is prolonged, and Cx43-ZO-1 conjugates that appear in GJ plaques affect the functioning of GJIC by always keeping the channel in an open state [25]. Phosphorylation of Cx43 at S373 inhibits its binding, whereas phosphorylation at S365 does the opposite [25, 69]. The opening of GJ gates is also influenced by many factors. Modification of Cx43 by cAMP/protein kinase A (PKA) signaling promotes the opening of GJs. Ezrin can furthermore assist in the binding of Cx43 to PKA or ZO-1 and is necessary for Cx43 phosphorylation and GJIC [70, 71]. In addition, β-catenin promotes the stabilization of GJs with the Wnt/β-catenin pathway promoting Cx expression [72]. The localization of NF-κB also promotes *GJA1* expression [73].

#### Negative regulation of Cxs and GJs

Negative regulation of Cxs or GJs generally leads to the degradation of both or the closure and internalization of the latter. Upon synthesis in the endoplasmic reticulum, Cxs may be degraded by proteasomes in a ubiquitin-dependent manner as modulated by CIP75 [61]. GJ plaques located at the junction of two cells depress unilaterally to form connexosomes in a process called internalization [26]. Connexosomes are degraded directly via lysosomes or autophagy [74–78]. They can also enter the early endosome and late endosome before finally being degraded via lysosomes [79, 80]. As mentioned above, the ubiquitination of Cxs, as well as phosphorylation at some sites, can promote the internalization and degradation of GJs. Notably, protein kinase C (PKC) can be activated by vascular endothelial growth factor (VEGF), an exchange protein that is directly activated by cAMP [81]. Modification of Cx43 by mitogen activated protein kinase (MAPK) promotes its binding to Nedd4 [81, 82]. The Cx43 binding region for Nedd4 is also the binding site for tumor susceptibility gene 101 and the AP2 adaptor protein complex. These proteins promote the degradation of GJs [79, 83]. Epidermal growth factor promotes the endocytosis of Cx43 through the MAPK and PKC pathways. Modification of the Tyr247 and Tyr265 sites of Cx43 by Src blocks Cx43 from binding to tubulin. Cx43 downregulation increases focal adhesion kinase-Src activation [84, 85]. The conjugates of these two proteins are early regulators of integrin signaling and can promote the invasion and metastasis of tumor cells [85]. c-Src regulates EMT via the PI3K/Akt pathway. A reduction in the ability of c-Src to combine with Cx43 increases the activity of Akt, thus enhancing the ability of cancer cells to invade and metastasize [86].

#### PTMs of Cxs

PTMs are of great significance for the stability and function of Cxs, in which they occur via phosphorylation, ubiquitination, acetylation, and SUMOylation of the proteins. The carboxyl termini of different Cx isoforms vary substantially and comprise the major sites of PTMs. Modification by phosphorylation changes the half-life of Cxs as well as their ability to form GJs and resulting gating properties [71, 87]. Ubiquitination, acetylation, and SUMOylation mainly regulate Cx or GJ degradation.

#### Phosphorylation of Cxs

**Fig. 2:**
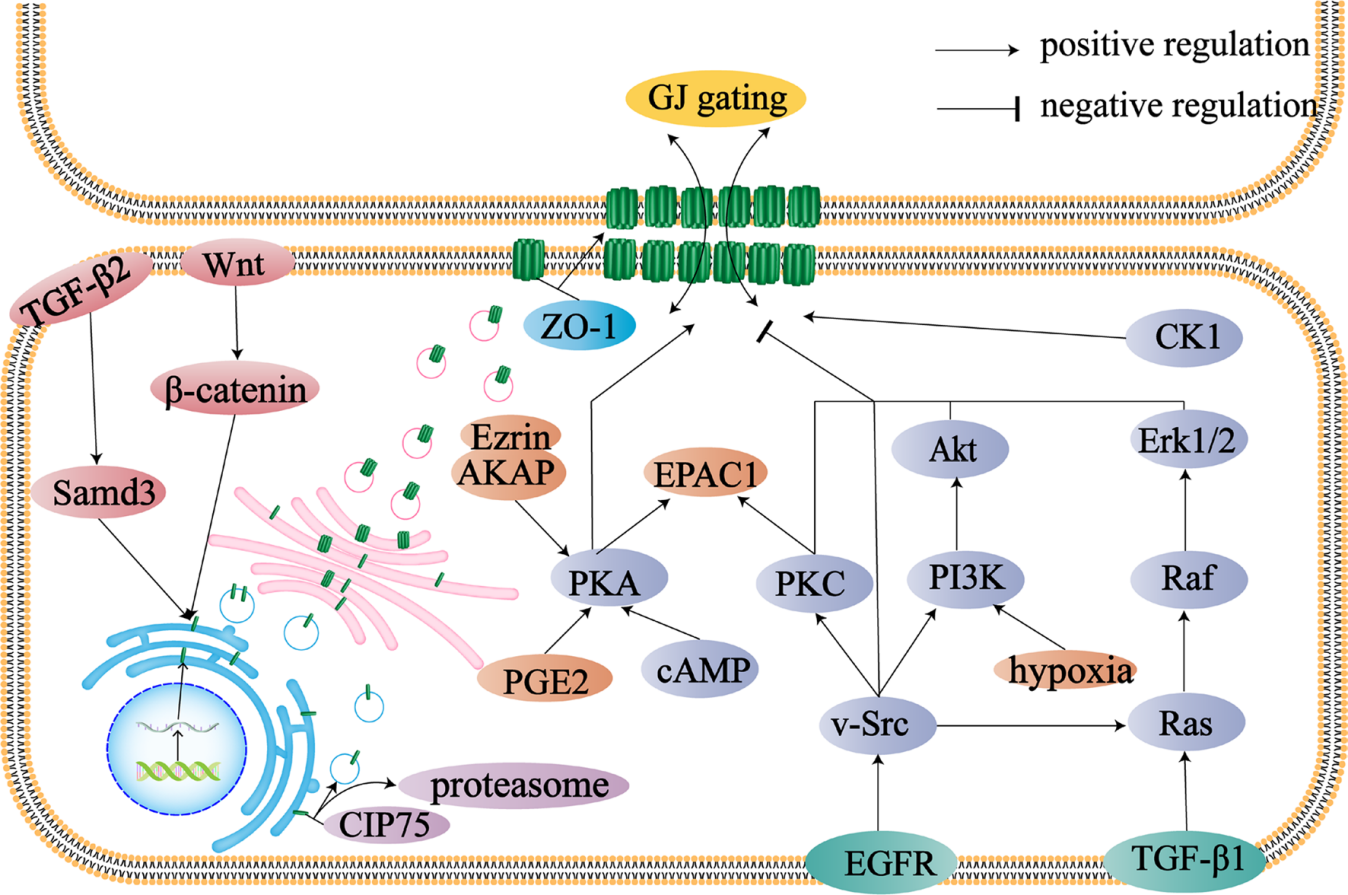
Schematic diagram of the regulation of Cx synthesis and assembly. The formation and assembly stages of Cxs are influenced by various kinases and chaperones. In this figure, Cx43 is used as an example to describe the factors for Cx expression, formation of GJs, and gating regulation

Modification of Cx phosphorylation is closely associated with the malignant progression of cancer. Gould et al. showed that in situ and invasive breast carcinoma tissues exhibit more Cx43 phosphorylation than normal breast tissues [88]. Phosphorylation of Cx43 also contributes to the development of pancreatic cancer [89]. In addition, phosphorylation of Cxs may function as a prognostic marker for gliomas [90]. Dysregulation of Cx phosphorylation and restoration of normal GJIC or GJs in cells may be potential targets for cancer therapy [91, 92]. Malignant gliomas show increased Cx phosphorylation [90]. Phosphorylation of Cx43 at Ser279 has been shown to promote tumor vessel formation [93]. Phosphorylation of Cxs is associated with the number of GJs, dynamic changes in GJ plaques, and the internalization of GJs [94, 95]. The enzymes capable of phosphorylating Cxs mainly include PKA, PKC, MAPK, casein kinase 1 (CK1), and Src (Fig. 2). Most phosphorylation sites of Cxs are located at their carboxyl termini, and different modification sites have different effects on GJIC. Phosphorylation of Cxs leads to not only the localization of GJs on cytomembranes, but also the formation of hemichannels on cyto- and mitochondrial membranes.

Cx43 is the most commonly expressed Cx. The potential phosphorylation sites of PKA in Cx43 include Ser364, Ser365, Ser368, Ser369, and Ser373. Phosphorylation of Cx43 by PKA can promote GJ assembly and GJIC function [71] and reduce the migration ability of cells [96]. Notably, the Ser365 site, which can hinder PKC modification at Ser368, has a protective effect on GJs [97]. Akt, the downstream target of PI3K, mainly modifies the Ser373 and Ser369 sites in Cx43 [98]. These sites are modified to increase Cx43 cell membrane localization and to promote gate opening. Geletu et al. [99] found that PI3K may be necessary for GJ formation. The modification of Cx43 by Akt reduces Cx43 binding to 14-3-3 protein [100]. The 14-3-3 protein can facilitate the trafficking of Cx43 from the cytoplasm to the cell membrane and its assembly into GJs [100]. ZO-1 is one of the chaperone proteins of Cx43 that can promote the formation of GJs and maintain the open state of GJ gating and stability of GJ plaques [25, 69]. However, the ability of Cx43 to bind to ZO-1 diminishes after its S373 site is phosphorylated. PKC acts as a downstream target for phospholipase C. However, phosphorylation of the Ser368 site of Cx43 by PKC causes the closure of or a reduction in the number of GJs, which is not conducive to cell-to-cell signal delivery and material communication [94, 101]. Phosphorylation of Cx43 at Ser368 is important for the cell cycle. Phosphorylation at this site is generally elevated in the S and G2/M phases of mitosis [102]. MAPK mainly modifies the Ser255, Ser262, Ser279, and Ser282 sites of Cx43, and GJIC is inhibited after phosphorylation at these sites [81]. The phosphorylation of these sites by MAPK also affects the binding of Cx43 to cyclin E, which may lead to an increase in the cell proliferation ability [103]. During the prophase and metaphase of mitosis, a smaller proportion of Cx43 is involved in the formation of GJs, and numerous GJs are internalized. When mitosis enters the telophase, a large number of GJ plaques appear between cells [104]. This may be related to the modification of Ser255 and Ser262 sites of Cx43 by CDC2 [105, 106]. CDC2 levels may not significantly fluctuate during the cell cycle; however, its activity peaks in the G2/M phase. Notably, *SRC*, an oncogene, can not only directly modify Tyr247, Tyr265, and Tyr313 of Cx43 (which also inhibits or even destroys GJs) but can also indirectly modify Cx43 and further affect GJIC through PKC and Erk1/2, Ras/Raf/Erk, and PI3K/Akt [107–110]. The phosphorylation of Cx43 by CK1 regulates GJ assembly. A study on pancreatic ductal adenocarcinoma by Solan et al. confirmed that GJs are abundant in stromal cells of Kras (LSL-G12D/+; p48Cre/+) mice but absent in KC; Cx43 (CK1A) mice (crossing the Cx43(CK1A) mouse onto the KC background), and that KC;Cx (CK1A) cells can efficiently downregulate Cx43 expression [89]. Combining CX43 with C-terminal Src kinase and phosphatase and tensin homolog in the region between residues 266 and 283 within the C-terminus can inhibit Src activity [111].

As a proto-oncogene, the tyrosine-protein kinase, Src, regulates the phosphorylation of Cxs, allowing Cx internalization and inhibiting GJIC. The phosphorylation level of Cxs increases the malignancy of breast cancer cells [88]. The Src effector, STAT3, may be necessary for GJs to perform their functions. The number of GJs does not change appreciably following treatment of cells with STAT3 inhibitors; however, their permeability decreases [112]. Other types of Cxs such as Cx26, Cx32, Cx36, and Cx45 have also been confirmed to be phosphorylated. The same protein kinases differ with regard to the modification sites of different Cxs; the main sites modified by PKA in Cx35 are Ser110 and Ser276. PKA inhibits GJIC function in a Cx35-dependent manner [113].

#### Ubiquitination of Cxs

Ubiquitination is another important process that forms PTMs of proteins and has a huge impact on protein function. Several studies have demonstrated that ubiquitination is associated with the internalization and degradation of GJs. The lysine residues of Cx43 are important sites for ubiquitination. Ubiquitination of Cx43 is a reversible process that can be modulated by many molecules to ensure the stability of Cx43 at the cell membrane. For example, Nedd4 [82], epidermal growth factor, and ginsenoside Rg-1 [114] promote ubiquitination. However, molecules associated with the SH3 domain of the signal transducing adaptor molecules (a subunit of the endosomal sorting complex required for transport-0) [115] and ubiquitin-specific peptidase 8 [116] can downregulate the ubiquitination level of Cx43. Ubiquitinated Cx43 is degraded through different pathways, including the proteasome and lysosomal pathways, and autophagy [80, 117, 118]. The entry of Cxs into different degradation pathways may be related to their subcellular localization. Ubiquitination of Cx is not isolated from phosphorylation; they are interrelated. One study showed that high phosphorylation of Ser368, Ser279, Ser282, and Ser255 sites occurred when ubiquitination of Cx43 Lys264 and Lys303 sites was inhibited [119]. This phenomenon suggests that phosphorylation can induce ubiquitination. Growing evidence from further research suggests that the crosstalk between phosphorylation and ubiquitination of Cxs can regulate the internalization of GJs and degradation of Cxs. The ubiquitination and de-ubiquitination of Cx32 and Cx40 are tightly regulated [120, 121]. Mitra et al. showed that prostate carcinogenesis may be accompanied by the dysregulation of Cx degradation [122]. In addition, an increase in the degradation of Cxs is generally accompanied by a decrease in the number of GJs. This may result in a decrease in the sensitivity of cancer cells to chemotherapeutic drugs and a weakening of radiation-induced bystander effects [11, 123].

#### SUMOylation of Cxs

SUMOylation is a PTM which, in proteins, is a process in which small ubiquitin-like modifier (SUMO) proteins bind to substrates covalently or non-covalently under the catalysis of SUMO proteases [124]. In contrast to ubiquitination, SUMOylation can have a positive effect on the stability of Cx43 at the cell membrane [125]. SUMOylation regulates the stability of Cxs. Upregulation of SUMO protein expression can improve GJIC, which enhances information exchange between tumor cells [125–127]. The SUMO system is associated with Cx43 protein levels, and SUMO 1/2/3 at Lys 144 and Lys 237 can modify Cx43. Mutations in lysine 144 or lysine 237 lead to a reduction in Cx43 protein and GJ levels [125]. Modification of Cx43 by SUMO 1 improves GJIC function in hepatocarcinoma cancer stem cells (CSCs), and further experiments have confirmed that this improvement has a positive effect on the sensitivity of tumor cells to herpes simplex virus thymidine kinase/ganciclovir HEMC [126]. SUMO 2 and SUMO 3 have been shown to equally promote the formation of GJs on cell membranes, in addition to SUMO 1 [125]. Ubiquitination acts opposite to SUMOylation, and the internalization and degradation of Cxs can be effectively controlled. Osteosarcoma cells highly express the SUMO-conjugating enzyme, Ubc9. A decrease in Ubc9 expression can inhibit osteosarcoma cell proliferation and migration and induce decoupling of SUMO 1 from Cx43, increasing free Cx43 levels [127].

#### Acetylation of Cxs

The sites of acetylation in Cxs are also mainly located at the carboxyl termini. In vitro studies have shown that acetylation of Cx32 enhances the proliferative capacity of cells but does not affect the function of Cx32-dependent GJs [128]. Accumulation of Cx32 in cells after acetylation can increase cell proliferation; however, the mechanism underlying this process remains unclear [128]. Cx43 can also be acetylated. The localization of Cx43 to the cell membrane reduces after acetylation. The binding of Cx43 to ZO-1 is blocked after acetylation, but the binding to Src is not compromised [129]. Interestingly, acetylation and ubiquitination may also be interrelated, as the level of ubiquitination tends to decrease as the degree of Cx32 acetylation increases [128]. Unlike Cx32, other types of Cxs have not been found to exhibit such crosstalk.

Other modifications of Cxs, such as O-GlcNAc glycosylation [130], nitrosylation [131, 132], carboxylation, glutamate γ-carboxylation, and methylation [133], are also important for their stability and membrane distribution. PTMs have significant effects on the permeability of GJs and the stability and subcellular localization of Cxs. Cancer development is usually accompanied by changes in the PTM of Cxs [88], which may weaken the body's control over cancer cells, block the spread of anti-cancer drugs among cancer cells, and increase drug resistance in cancer cells. The efficiency of synthesis and degradation of Cxs and GJs (which governs their numbers) have a crucial impact on cancer cell behavior such as migration ability and cell cycle progression [89, 96, 134]. An in-depth exploration of the PTM of Cxs may provide novel insights into methods for curbing cancer progression.

### GJs and cancer

#### GJs regulate tumorigenesis

Normal epithelial cells exhibit cell polarity in vivo, which is important for maintaining epithelial cell stability and contributes to the coordination of intracellular functions. A critical pattern of cell polarity within epithelial cells is the apical-basal polarity [135]. Studies have shown that disruption of epithelial cell polarity can be regarded as an indicator of cancer development [136]. Polarized microtubules are essential for cell polarity. Bazzoun et al. found that GJs are involved in polarity formation in breast epithelial cells. Disruption of GJ function may lead to the loss of homeostasis in epithelial cells and eventually tumorigenesis [137]. In addition, in the initial stages of tumorigenesis, functional GJs can help cells dedifferentiate and acquire immortality by modifying telomerase [138]. Decreased expression and altered subcellular localization of Cxs occur in many types of malignant tumors, including those of gastric cancer [139], non-small cell lung cancer [140, 141] (NSCLC), and bladder cancer [142].

Cx43 can bind to microtubules and be transported to the cell membrane, and this process maintains the stability of microtubules [26, 143]. The absence of microtubules prevents the development of cell polarity. Upon cancer initiation, the expression of Cx43 decreases, which may be associated with the destruction of cancer cell polarity [137, 143]. The subsequent series of signaling pathways initiated by integrins are also important for the maintenance of the apical-basal polarity of epithelial cells [135]. Downregulation of Cx43 can change the subcellular distribution of integrins, thereby affecting the cytoskeleton and cell polarity [144]. Endothelial cells, which are specialized epithelial cells, also exhibit polar characteristics. The binding of Cx43 to vasodilator-stimulated phosphoproteins in human vascular endothelial cells is of positive significance for the directional migration of cells and maintenance of cell polarity [96]. In addition, GJB2 knockdown increases the probability of carcinogenesis in breast cancer [145]. In addition, decreased expression of Cx26, compared to that in normal breast tissue, or even no expression, has been found in breast cancer [146].

#### GJs can promote tumor angiogenesis

Tumor angiogenesis is important for tumor metastasis and rapid growth [147]. Heterogeneous communication between cancer and vascular endothelial cells is essential for tumor angiogenesis [148]. Co-culture of metastatic colorectal cancer cells with human microvascular endothelial cells (HMECs) can promote Cx phosphorylation and alter Cx subcellular localization from the cell membrane to the cytosol [148]. The effects of GJIC between HMECs and cancer cells cause transendothelial cell metastasis and tumor angiogenesis [148]. Inhibition of tumor angiogenesis by regulating GJIC or Cxs may become a target for anti-tumor therapy in the future. Thuringer et al. [8] demonstrated in vitro that miRNA-145 can be transmitted between HMECs and SW480 colon cancer cells. Transmission of miRNA-145 from HMECs to SW480 cells increases Cx43 expression in cancer cells. It also reduces the ability of cancer cells to promote angiogenesis. In addition to miRNA-145, miR-30b and miR-200b affect tumor angiogenesis in a specific manner with the participation of Cxs [149]. Many researchers believe that an increase in Cx43 levels disrupts the integrity of tumor blood vessels [151,152]. Murine xenografts from Cx43-overexpressing cells have fewer and smaller blood vessels than those from wild-type cells [152,153]. VEGF promotes vascular endothelial formation and injury repair through Cx43- and Cx43-dependent GJIC [154]. Prevention of VEGF binding to its receptor and inhibition of GJIC can arrest the breast cancer cell line MDA-MB-231 in the G1/S phase and inhibits the proliferation, invasion, and metastatic ability of the cells to varying degrees [155]. Xenografts from MDA-MB-231 cells receiving oleamide and avastin (inhibitors of GJIC and VEGF, respectively) treatment exhibit smaller tumor masses and reduced liver and lung metastases than those without treatment [155]. Fukuda et al. [156] found that homogenous communication between cancer cells had less of an effect on tumor progression than heterogeneous communication between cancer cells and normal tissues. Cx37 and Cx40 form intercellular junctions between endothelial cells and participate in developmental angiogenesis. Loss of Cx37 and Cx40 inhibits angiogenesis and decreases the growth of malignant tumors, suggesting that these Cxs may be targets for anti-tumor treatments [157].

#### GJs participate in regulating the cell cycle of cancer cells

Many studies have shown that either GJIC or Cxs participate in the regulation of cancer cell growth. The regulation of the cell cycle by Cxs mainly depends on their interactions with cell cycle regulators [2, 3]. Increased Cx expression prolongs the G1 phase. When the expression of Cx37 increases in insulinoma cells, the cell proliferation cycle becomes longer and remains at the G1/S boundary, weakening the proliferative ability of tumor cells [158]. Removal of growth factors during cell culture makes this phenomenon more obvious. In gastric cancer, Cx32 changes or even loses its subcellular localization with increasing malignancy [159]. In addition, the proportion of AGS gastric cancer cells in the S phase increases. Upregulation of Cx32 expression can also increase the proportion of cancer cells remaining in the G1 phase [160]. Cx32 affects the cell cycle probably because it promotes the expression of P21cip1 and P27kip1 [3]. GJs can also control the cell cycle by regulating the transmission of Ca^2+^ and other cellular factors [161]. In addition to their involvement in cell cycle regulation, the effects of GJs and Cxs on apoptosis cannot be ignored. GJIC can regulate cell death by delivering pro-death or anti-death factors [162]. Mitochondria play an important role in apoptosis. Although a decrease in Cx43 expression in mitochondrial membranes has been shown to promote apoptosis, the specific mechanism remains unclear [163]. Interestingly, tumors do not appear to be sensitive to mitochondria-induced apoptosis. The mechanism by which Cxs affect mitochondrial membranes and tumor apoptosis is also unknown.

#### GJs and cancer metabolism

There are significant differences in metabolism between cancer and normal cells. It is well known that the metabolism of most cancer cells mainly follows the Warburg effect, i.e., cancer cells prefer aerobic glycolysis to the tricarboxylic acid cycle to produce adenosine triphosphate (ATP) [164]. GJIC induces metabolic coupling between hypoxic and oxygen-rich cells in tumors [165], which can be used as a pathway to diffuse hypoxic cell metabolites such as lactate into oxygen-rich cells and, in turn, transfer HCO_3−_ from oxygen-rich cells to hypoxic cells [165]. Cells with higher low-glucose tolerance selected from the MDA-MB-231 cell line generally exhibit higher levels of Cx43 and GJs [166]. In a 3D culture of pancreatic cancer cells, lactate was transferred from the central hypoxic site to the normoxic boundary via GJs [167]. Inhibition of Cx43 expression in cancer cells significantly reduced lactate delivery to the boundary [167]. GJIC was also found to promote glucose trafficking to central hypoxic cells in colorectal cancer cells cultured using 3D stereo culture methods [168]. GJIC can promote cancer growth when there is a lack of tumor blood vessels for substance exchange [168]. Interestingly, in a co-culture of NSCLC cells with CAFs, we found unidirectional and heterogeneous GJIC from CAFs to NSCLC cells. Unidirectional GJIC inhibited glycolysis in NSCLC cells but increased mitochondrial oxidative phosphorylation. Moreover, CAFs showed enhanced glycolysis [6]. CAF metabolites can be transported to NSCLC cells via unidirectional GJs. These products are reused by NSCLC cells to generate more energy, which supports their activity. Eventually, CAFs promote the invasion and metastasis of NSCLC cells through GJs [6]. As a critical proto-oncoprotein, Cx45 is essential for the high concentrations of glucose needed to stimulate the proliferative capacity of liver cancer cells [1]. In BALB/c immunodeficient mice of varying blood glucose concentrations inoculated with human hepatoma cells, an increase in blood glucose concentration was accompanied by a corresponding increase in Cx45 expression and rapid growth of the tumor mass. Knockdown of Cx45 could abrogate the rapid growth of tumors stimulated by D-glucose in mice [1]. These results demonstrate that GJIC can also form a metabolic crosstalk with other stromal cells to meet their nutritional requirements.

#### GJs regulate tumor cell invasion and migration via EMT-dependent and independent pathways

EMT refers to the transformation of epithelial cells into cells with a mesenchymal phenotype via a program that enhances cell invasion and migration. EMT is generally associated with a decrease in E-cadherin levels and an increase in the expression of mesenchymal markers, including N-cadherin and vimentin [169]. This process results in a loss of cell polarity and cell-to-cell contact [169]. The co-localization of Cxs with E-cadherin or N-cadherin has been detected in many types of cancer cells [4, 5]. A reduction in E-cadherin levels reduces the adhesion ability of cancer cells, rendering them more likely to invade surrounding tissues across the basement membrane and to metastasize to distant sites. Cxs are involved in the regulation of EMT either as monomers or in a GJIC-dependent manner. Cx31.1 and Cx43 inhibit EMT in NSCLC [170], and Cx43 can reverse EMT in A549 lung adenocarcinoma [171]. A549 cells overexpressing Cx43 increase the expression levels of E-cadherin and decrease cellular invasive and migratory abilities [171]. In contrast, decreased expression of Cx43 promotes the metastasis of MDA-MB-231 cells. Cx26 interacts with endothelial cells and is involved in tumor cell intravasation and extravasation [172]. In the tumor microenvironment, GJIC between cancer and stromal cells generally promotes EMT. However, GJs can also facilitate metastasis in an EMT-dependent manner. The degree of EMT has been significantly enhanced by Cx26 overexpression in NSCLC cells [173]. Lastly, GJs can enhance cancer cell invasion and migration via an EMT-independent pathway. The expression of GJs between metastatic cancer cells and astrocytes is associated with brain metastasis of lung and breast cancers. GJIC occurs between CAFs and tumor cells; after the formation of a functional link between stromal and NSCLC cells through GJIC, CAFs differentiate into myofibroblasts and E-cadherin expression in cancer cells decreases, whereas N-cadherin expression increases [6].

Table 2 summarizes the varying effects of different connexins on EMT in breast, lung, and liver cancer. Even the effect of the same connexin isoform may differ among tumor types.

**Table 2:** The effect of Cxs regulation on EMT-related protein expression in different cancers

Cancer	Cx	Cell line	EMT	References
Breast carcinoma	Cx32	MDA-MB-231	promote	[174]
		Hs578T	inhibit	[175]
	Cx43	MDA-MB-231, T47D	inhibit	[86, 176]
	Cx46	MCF-7	promote	[177]
Pulmonary carcinoma	Cx26	HCC827, PC9	promote	[173]
	Cx31.1	H1299	inhibit	[170]
	Cx43	A549	inhibit	[171]
Hepatocellular carcinoma	Cx32	HepG2, SMMC-7721	inhibit	[178, 179]

#### GJs and cancer dormancy

Tumor cells enter a dormant phase for several reasons, including immune escape and chemoradiotherapy resistance. During clinical treatment of breast cancer, breast cancer cells (BCCs) enter the dormant phase; this suppressed tumor state is closely related to GJs. The main dormancy site of BCCs is the bone marrow (BM) [180]. After entering the BM, BCCs can be linked to the BM stroma through GJs. Some substances that maintain dormancy, such as miRNAs, can enter BCCs and render them quiescent at the G0 phase [181]. Alternatively, M2 macrophages present in the BM stroma can maintain the dormant/quiescent state of BCCs via GJs [182]. M1 macrophages are also present in the BM stroma. The effect of these cells on cancer dormancy is different from that of M2 macrophages in reversing the dormant state of BCCs [182]. After entering dormancy reversal, BCCs experience increased internalization of GJs and decreased GJIC and spread to other parts of the body, causing tumor recurrence.

#### GJs and cancer stem cells

CSCs, also known as tumor-initiating cells, are a special subset of cancer cells that accounts for only 1–2% of cancer tissues and play a crucial role in tumor recurrence and chemoradiotherapy resistance. The expression of Cxs in CSCs is divided into three categories: no Cxs expression, Cxs expression without GJIC function, and Cxs expression with GJIC function. Cx expression is often absent in CSCs of glioma and pancreatic cancers. Glioma CSCs possess a low expression of Cx43 to maintain the malignant phenotype, and restoration of Cx43 expression results in a reversal of the malignancy by modulating E-cadherin [183]. Cx26 is present in triple-negative breast CSCs in a GJ-independent manner, and its expression level here is significantly higher than in common tumor cells [7]. Cx26 acts synergistically with NANOG transcription factor and focal adhesion kinase in the nucleus to maintain stemness and self-renewal of breast CSCs [7]. Cx32 is present in the cytoplasm of liver CSCs in a GJ-independent manner. In lung cancer, overexpression of Cx43 decreases the abundance of CSCs and reduced proliferation, invasion, and metastasis ability [184]. Additionally, the effect of Cxs or GJs on maintaining self-renewal has also been demonstrated in embryonic and somatic stem cells. Secreted negative growth regulators from terminally differentiated daughters of stem cells or secreted stroma-derived factors can regulate the growth of stem cells without functional GJIC [185, 186]. The degree of CSC features in pancreatic cancer and gemcitabine resistance is associated with dysfunctional GJIC due to low or absent Cx43 protein levels [9]. However, an accumulation of cytoplasmic Cx32 increases the self-renewal of CSC in hepatocellular carcinoma [187]. Glioblastoma CSCs express higher levels of the GJs protein Cx46 than non-CSCs, and Cx46 maintains the proliferation and self-renewal of glioma CSCs [188]. Breast CSCs use Cx43 to form GJIC with BM niche cells, fibroblasts, and mesenchymal stem cells. Cx43 and N-cadherin conjugates have been found in abundance in breast CSCs [189].

### GJs are associated with cancer treatment

#### GJs and radiotherapy

GJIC is widely believed to have a positive effect on tumor therapy. Many different types of microwave beams used in clinical practice (e.g., X-rays and proton microbeams) cause damage to surrounding bystander cells in addition to radiated cells, a phenomenon called the radiation-induced bystander effect [11, 190]. Moreover, daughter cells of damaged bystander cells are subsequently affected [11]. One of the reasons for the bystander effect is that GJIC transmits substances that can cause cell damage, such as ROS produced by irradiated cells, to bystander cells [11]. ROS can trigger a series of DNA damage responses in cells, affect the cell cycle, and ultimately cause cell death. Inhibition of GJIC between bystander and irradiated cells reduces the damage to bystander cells and their daughter cells [11]. GJ-mediated cell-to-cell communication is usually bidirectional. Therefore, irradiated cells can affect bystander cells through GJs, and bystander cells can similarly transmit information to irradiated cells. Konishi et al. [191] confirmed that non-neoplastic bystander cells (WI-38) can promote DNA damage repair in irradiated cells via GJs. This differs from the previous view that GJIC is not involved in the protection of normal bystander and irradiated cells [192, 193]. Interestingly, the bystander effect, which involves GJIC, seems to protect surrounding normal bystander cells under hypoxia but is destructive to tumor cells [10]. This indicates that restoration of GJIC in hypoxic tumors may improve the accuracy of radiation therapy.

#### GJs and chemotherapy

Drug delivery between cells is important for therapeutic effects. In the initial stages of carcinogenesis, GJIC changes between cancer cells and between cancer and normal cells. Fluctuations in GJIC function may adversely affect the efficacy of anti-cancer drugs and cause cellular resistance. Forster et al. [9] conducted a controlled study using three pancreatic cancer cell lines with different degrees of resistance to gemcitabine: BxPc-3 (gemcitabine-sensitive), BxPc-3-GEM (resistant strain selected from BxPc-3), and AsPC-1 (gemcitabine-resistant). The more drug-resistant cell lines contained more CSC-like cells and exhibited the worst permeability of GJs. Platinum chemotherapy drugs are cytotoxic chemotherapeutic agents that can hinder DNA synthesis and replication, ultimately causing apoptosis. In vitro, cisplatin cytotoxicity positively correlates with the density of cancer cells. An increase in cell density enhances Cx43-based GJIC function and, ultimately, the rate of drug diffusion [123]. Additionally, oxaliplatin and cisplatin directly inhibit the expression of Cx43. A decrease in Cx43 expression not only inhibits GJIC function but also blocks drug diffusion between cells and decreases cytotoxicity [194]. In ovarian cancer, a reduction in cisplatin toxicity reduces Cx32 localization to the cell membrane and increases its cytosolic and nuclear expression [195]. It has been confirmed that cytosolic Cx32 inhibits cisplatin cytotoxicity [196]. Interestingly, Hong et al. [123] found that the effect of GJIC on normal cells was different from that on cancer cells when treated with cisplatin. GJIC reduces cisplatin-induced DNA damage in normal cells, but does not prevent cisplatin-induced cancer cell damage. However, the specific mechanisms underlying this phenomenon require further investigation.

The products of tumor suicide genes can convert prodrugs with little or no toxicity to the human body into toxic substances, eventually leading to cancer cell death [197]. The thymidine kinase (tk) gene, a suicide gene from the herpes simplex virus (HSV), converts ganciclovir (GCV) to toxic GCV-triphosphate [197]. GJIC-mediated bystander effects play important roles in the effects of HSV-tk/GCV. GJs deliver GCV-triphosphate compounds to other cancer cells. When GCV is used for cancer therapy, drugs such as all-trans retinaldehyde and histone deacetylation inhibitors enhance the function of GJIC and the cytotoxicity of HSV-tk/GCV [198]. Iron oxide nanoparticles [199], curcumin [200], dioscin [201], and resveratrol [202] also enhance the expression of Cxs and promote the cytotoxicity of HSV-tk/GCV.

Furthermore, Cxs can also mediate tumor resistance independent of GJIC. Gefitinib is a common targeted agent used in the treatment of NSCLC. Gefitinib-resistant NSCLC cell lines exhibit increased Cx26 expression in the cytoplasm and decreased membrane expression [173]. Moreover, the degree of EMT is aggravated in resistant cells. Cx26 may affect EMT through the PI3K/Akt pathway, but this process is not dependent on GJIC [173]. Resistance of glioblastoma to the commonly used chemotherapeutic drug, temozolomide (TMZ), is currently an urgent clinical problem. Decreased Cx43 expression renders glioma cells sensitive to TMZ [203]. Moreover, Yang et al. [204] found that TMZ-resistant cells secrete exosomes with higher Cx43 content than TMZ-sensitive cells; such high-content exosomes are more easily taken up by resistant cells. Cells that ingest these exosomes show greater metastasis, colony formation ability, and resistance to TMZ. Therefore, studies targeting exosomal Cx43 may provide new therapeutic strategies for glioblastoma treatment. Hemichannels composed of Cxs are also found in extracellular vesicle membranes. Such channels can transfer information to distant cells [205, 206]. This property can be exploited to deliver therapeutic drugs directly to target cells. Notably, Cxs are widely expressed in human cells and tissues; therefore, the targeted drugs for a certain subtype of Cx should be highly specific to avoid adverse effects on other subtypes of Cxs.

We mentioned here that a reduction in Cx43 expression can reduce cell sensitivity to cisplatin and oxaliplatin; however, a reduction in Cx43 expression can increase the sensitivity of malignant glioma cells to TMZ [207]. Many reports have indicated opposite effects of Cxs on cancer cell resistance. Moreover, different Cxs have different effects on chemoresistance in different tumor types. The pathway or mechanism by which Cxs mediate chemoresistance requires further investigation.

#### GJs and other cancer treatments

In addition to radiotherapy and chemotherapy, the emerging nanosecond pulse tumor ablation technology in clinical treatment is associated with GJIC. A pulsed electric field induces the internalization of Cx43 and inhibits the expression of Cx43 and its mRNA [208]. In addition, kinases such as MAPK, which can modify Cxs, are activated in the presence of nanosecond-pulsed electric fields [208]. Hyperphosphorylated Cxs negatively affect the occurrence and function of GJIC. Photodynamic therapy (PDT) has been widely used in the treatment of malignant tumors such as esophageal cancer, lung cancer, and basal cell carcinoma [209]. Wu et al. [210] found that Cx43-dependent GJIC correlated with the effect of PDT. At high cell densities, an increase in intercellular Cx43-dependent GJIC leads to enhanced toxicity of luciferin, making malignant cells more sensitive to this compound. This phenomenon may be related to the transmission of ROS between cells via GJIC. It may also be related to the exchange of small molecules, such as Ca^2+^ and ceramide, between cells [210]. GJs composed of different types of Cxs have different effects on the efficacy of PDT. Wu et al. found that Cx32/Cx26-dependent GJIC reduced the sensitivity of malignant cells to phototoxins [211].

#### Small-molecule inhibitors targeting GJs

Cx-mimetic peptides are analogs of amino acid sequences corresponding to conserved regions of Cxs that can reversibly bind to Cxs to regulate GJIC. They can be subdivided into three classes: extracellular loop domain, cytoplasmic loop domain, and cytoplasmic carboxyl-terminal domain mimetic peptides. Gap26 is a mimetic peptide corresponding to the conserved region of the first outer loop of Cx43. In a study by Desplantez et al. [212], the gate-opening ability of Cx43-composed hemichannels reduced upon the treatment of HeLa cells with Gap26. Correspondingly, the permeability of GJs formed when the HeLa cells came into contact with each other also reduced. It is possible that Gap26 first acts on the hemichannel composed of Cx43 and then gradually affects the GJs. This effect of Gap26 is concentration-dependent [213]. Gap27 is an analog within the second outer loop region of Cx43 and acts in a manner similar to that of Gap26 [213]. Gap27 that is attached to a segment of the lipid tail can form a lipidated Cx-mimetic peptide. This novel mimetic peptide induces the phosphorylation of the Cx43 Ser368 site [214]. Mimetic peptides act not only on hemichannels in the cell membrane but also on those present in the mitochondrial inner membrane [214].

GJ channel opening requires contact between the carboxyl termini of Cxs and the cytoplasmic ring. The principle of action of cytoplasmic loop region-mimetic peptides (e.g., Gap19 and L2) is to regulate channel gating mainly by blocking the interaction of the Cx cytoplasmic loop with the carboxyl terminus [215, 216]. Since the action site of this kind of peptidomimetic is inside the cell membrane, it needs to be carried into the cell by cell-penetrating peptides such as TAT and Xentry [217]. In radiotherapy, substances that can cause cell damage or even death, such as ROS, can be produced in normal cells; these substances can also enter normal cells from other cells through GJs [218, 219]. The application of Gap19 (a mimetic peptide in the cytoplasmic loop region of Cx43) in vitro can decrease the permeability of membrane channels to protect normal endothelial cells. Endothelial cells were treated with TAT–Gap19 before irradiation. Compared with that in untreated cells, the amount of ROS in treated cells reduced significantly; hence, cell death due to radiation also reduced significantly [218]. TAT–Gap19 can enter the mitochondrial membrane, causing a decrease in the permeability of the mitochondrial inner membrane to Ca^2+^ and preventing cell death induced by Ca^2+^ entry [220].

The mimetic peptide, Cx43 266–283, corresponding to the cytoplasmic carboxy terminal domain of Cx43, can be carried by TAT from the cell membrane into the cytoplasm to take effect. In in vitro and in vivo experiments, TAT-Cx43 266–283 effectively slowed down the growth of malignant glioma cells and inhibited their invasion and metastasis by binding to the carboxyl terminus of Cx43 [221]. This effect is more specific, and normal astrocytes and neurons in the brain sections of mice are not affected [221], mainly because Cx43 266–283 can inhibit the interaction of c-Src with Cx43 [84, 221]. c-Src enhances the self-renewal ability of stem cells, whereas Cx43 inhibits c-Src activity [222, 223]. Another carboxyl-terminal region mimetic peptide, alpha-connexin carboxyl-terminal (ACT1), also shows great promise for cancer therapy. After the treatment of BCCs with ACT1, GJs showed an increased ability to transport substances. The proliferation ability of cancer cells reduced compared with that of normal breast epithelial cells in the control group [224]. Additionally, the combination of ACT1 with chemotherapeutic agents, such as tamoxifen and lapatinib, enhanced drug efficacy [224].

Table 3 briefly summarizes specific Cx inhibitors (peptidomimetics) along with their Cx targets and binding locations. In addition to specific Cx inhibitors, there are non-specific chemical Cx inhibitors, such as octanol and glycyrrhetinic acid and its analogs. These inhibitors are thought to dissolve in the lipid bilayer of the cell membrane, altering the local fluidity of the membrane and ultimately affecting GJ channels [225].

**Table 3:** Mimetic peptides that target connexins (Cxs)

Cx target	Peptide	Sequence	Location
Cx43	Gap26 [212]	VCYDKSFPISHVR	EL1
	Gap27 [226]	SRPTEKTIFII	EL2
	Gap36 [227]	KRDPCHQVDCFLSRPTEK	EL2
	Peptide 5 [228]	VDCFLSRPTEKT	EL2
	Gap19 [229]	KQIEIKKFK	Cytoplasmic loop
	L2 [230]		Cytoplasmic loop
	Cx43 266–283		Carboxy terminal
	ACT1		Carboxy terminal
Cx32	Gap27 [231]	SRPTEKTVFT	EL2
	Des5 [227]	LEGHGDPLHLEEC	Cytoplasmic loop
	Gap24 [232]	GHGDPLHLEEVKC	Cytoplasmic loop
Cx40	Gap27 [233]	SRPTEKNVFIV	EL2

This table briefly introduces peptidomimetics corresponding to various Cxs. The sequences and sites of action of the peptidomimetics are also listed.

### GJs and the prognosis of cancer patients

Tumors and Cxs are inextricably linked. Cxs not only affect tumor progression but their expression also provides indications for the prognosis of tumor patients. During the development of colorectal cancer, the function of GJIC is inhibited, and the expression of Cx43 and Cx32 gradually changes from strong membrane expression to cytoplasmic expression [234]. Cx43 can also be used as an independent prognostic marker for breast, oral squamous cell, and gastric carcinomas [235–239]. The mRNAs levels of several important Cxs (Cx26, Cx30.3, Cx32, and Cx43) in patients with NSCLC can be used to predict their overall survival rate. These Cx subtypes have different prognostic correlations with tumors and are more applicable to lung adenocarcinoma than lung squamous carcinoma [141]. The main reason for the fluctuation in mRNA levels during tumor development at different stages is the changes in the degree of DNA methylation. Therefore, some scholars have found that it is also feasible to use the changes in the Cx promoters, CpG islands, to predict the prognosis of gliomas [240]. Another surprising finding of this study was that nuclear Cx43 expression correlates with overall survival of patients with NSCLC.

Interestingly, Cx43 is often regarded as a tumor suppressor, whereas Cx26 has the opposite effect. In esophageal squamous cell carcinoma, the five-year survival of patients with a high expression of Cx26 is lower than that of patients with no or low expression, and high expression of Cx26 is often accompanied by lymph node metastasis [241]. In addition to being independent prognostic markers, Cxs can be used to predict the survival period of patients when considered in combination with other factors. Zhu et al. [242] found that endoplasmic reticulum oxidoreductase 1 beta, endoplasmic reticulum oxidoreductase 1-like beta, and Cx26 can jointly be used to predict the prognosis of pancreatic cancer; however, the samples selected for this experiment were all in situ carcinoma samples, the mechanism of which remains unclear.

## Conclusion

The importance of GJs in the body is self-evident; hence, any factor that can affect GJs, such as post-translational modifications, interactions with other proteins, and Cx peptidomimetics, are worthy of in-depth exploration. GJIC has a profound effect on the occurrence and development of cancer. GJs between normal and tumor cells can regulate cancer cells to a certain extent. If such GJs decrease in number or are completely lost, the malignancy of cancer cells increases, enhancing their ability to invade and metastasize. GJs can promote the cytotoxicity of various chemotherapeutic drugs. Upregulating the number of GJs in cancer cells appropriately can alleviate drug resistance. The widespread bystander effect in radiotherapy is also inseparable from GJIC. Some substances that can damage normal cells, such as ROS generated during chemotherapy, can be transmitted to bystander cells through GJs, causing negative effects.

GJIC is a double-edged sword. The goal of scientific research on GJIC is to use GJs or Cxs to develop novel cancer treatment methods. There is still a long way to go for cancer research and treatment, and Cxs or GJs (which are composed of Cxs) may become new therapeutic targets. As Cxs and GJs are widespread in tissues, some treatments targeting them may have significant side effects in humans. Therefore, there are currently no drugs targeting GJs for use in clinical settings.

## Data Availability

No available.

## Abbreviations

GJs: Gap junctions
Cxs: Connexins
GJIC: Gap junction intercellular communication
CAFs: Cancer-associated fibroblasts
EMT: Epithelial–mesenchymal transition
ROS: Reactive oxygen species
PTMs: Post-translational modifications
miRNA: MicroRNA
CIP75: Connexin 43-interacting protein of 75 kDa
ZO-1: Zonula occludens-1
PKA: Protein kinase A
PKC: Protein kinase C
VEGF: Vascular endothelial growth factor
MAPK: Mitogen activated protein kinase
CK1: Casein kinase 1
SUMO: Small ubiquitin-like modifier
CSCs: Cancer stem cells
NSCLC: Non-small cell lung cancer
HMECs: Human microvascular endothelial cells
BCCs: Breast cancer cells
BM: Bone marrow
HSV-tk/GCV: Herpes simplex virus thymidine kinase/ganciclovir
TMZ: Temozolomide
PDT: Photodynamic therapy
ACT1: Alpha-connexin carboxyl-terminal
